# Beyond the streetlight: a TREAT‐AD perspective on where to find new Alzheimer's targets

**DOI:** 10.1002/alz.71142

**Published:** 2026-02-13

**Authors:** Gregory A. Cary, Jesse C. Wiley, Gregory W. Carter, Allan I. Levey

**Affiliations:** ^1^ The Jackson Laboratory Bar Harbor Maine USA; ^2^ Department of Pharmacology & Toxicology University of Kansas Lawrence Kansas USA; ^3^ Goizueta Brain Health Institute and Alzheimer's Disease Research Center Emory University Atlanta Georgia USA

**Keywords:** Alzheimer's disease, biological domain, clinical trial

## Abstract

Despite extensive investments in Alzheimer's disease (AD) therapeutic development, progress toward effective interventions remains modest. The landscape of potential novel therapeutic strategies is rapidly growing, but prioritization, validation, and tools to advance targets to trial are lagging. The Target Enablement to Accelerate Therapy Development for Alzheimer's Disease (TREAT‐AD) consortium has integrated systems‐level data from large‐scale studies profiling thousands of human brains, yielding target‐specific risk scores that partition disease risk into discrete biological domains and enable data‐driven target interrogation. Here, we compared clinical trial targets with top‐ranked TREAT‐AD targets and found a limited overlap as well as differences in the biology emphasized by each set. The current AD therapeutic development landscape remains largely under the “streetlight” of familiar biology, while unbiased measures of disease risk point toward other disease‐associated processes that remain comparatively underexplored. These findings underscore opportunities to more deliberately diversify therapeutic portfolios and complement existing development efforts with evidence derived from unbiased human data.

**Highlights:**

- Clinical AD trials remain focused on well‐characterized biology.
- TREAT‐AD integrates genetic and multi‐omic data to prioritize novel targets.
- Limited overlap exists between clinical and high‐risk data‐driven targets.
- Risk‐associated targets uniquely implicate mitochondrial, lipid, and other pathways.
- Advancing “dark” targets is critical to diversify AD therapeutic strategies.

## 1 BACKGROUND

Alzheimer's disease (AD) is a devastating, progressive neurodegenerative disorder. With aging populations, AD prevalence is projected to increase in the coming decades, exacerbating the already immense emotional and economic burden on patients, families, and healthcare systems.^1^ There exists an urgent need to rapidly identify therapeutic approaches that will alleviate these burdens. Recent decades of concerted efforts to develop disease‐modifying therapeutics have been marked by very high rates of clinical trials failing to meet primary endpoints. However, recently approved anti‐amyloid therapeutics demonstrate that, despite modest clinical benefits and high‐risk side effects, disease modification is an achievable goal. These successes have redoubled the emphasis on the need for a diverse portfolio of clinical interventions that address the full range of disease pathology. In practice, AD and related dementias (ADRD) involve a mix of pathologies – accumulation of amyloid beta (Aβ) and tau, cerebrovascular disease, neuroinflammation, endolysosomal and proteostasis dysfunction, metabolic dyshomeostasis, and synaptic degeneration – whose contributions vary across individuals and stages. These realities argue for a diversification of therapeutic mechanisms under investigation.

The AD therapeutic development pipeline has been diversifying in recent years, with nearly twice as many trials and drugs in the Phase I pipeline in 2025 compared with 2024.^2^ The targeted mechanisms in these trials have been categorized using the Common Alzheimer's Disease Research Ontology (CADRO), and though nearly one‐third of interventions in the development pipeline are still focused on addressing the core pathologies (Aβ and tau), there are also agents emerging that target other therapeutic categories including inflammation, neurotransmitter receptors, synaptic plasticity and neuroprotection, vasculature, growth factors and hormones, and proteostasis/proteinopathies.^2^ However, the potential therapeutic landscape is far broader, as the highly complex underpinnings and heterogeneity of AD and ADRD are only recently coming to light. A rapidly expanding diversity of trial targets is being driven by the influx of systems‐level molecular analyses of AD, including genome‐wide association studies (GWASs) and other large‐scale omics studies of human tissues (e.g., proteomics, transcriptomics), which implicate hundreds to thousands of candidate molecular targets based on disease association. The Accelerating Medicines Partnership Program for Alzheimer's Disease (AMP AD), for example, has established a new model for collaborative, early‐stage target identification, generating omics data from multiple modalities across several thousand biosamples obtained from several clinically and pathologically phenotyped cohorts, with data made openly available on the AD knowledge portal.^3^ These data represent unbiased, genome‐wide measures of gene, transcript, metabolite, and protein involvement in AD. These approaches complement rather than replace existing hypothesis‐driven efforts, providing an orthogonal view of disease‐associated biology. This accessibility has catalyzed independent validation, computational innovation, and integration efforts such as the Target Enablement to Accelerate Therapy Development for Alzheimer's Disease (TREAT‐AD) consortium. TREAT‐AD has integrated these data and summarized the risk burden for each gene product in the genome.^4^ A primary goal of these efforts is to mine the wealth of information emanating from patient cohort studies to facilitate the nomination and validation of promising data‐driven targets and develop research tools to enable early‐stage therapeutic campaigns.

Given the availability of systematic, comprehensive assessments of AD risk, it is worth asking whether this wealth of genetic and genomic knowledge is being effectively translated to the clinical development pipeline. We systematically compared the targets of agents currently in the clinical trial pipeline for AD with the targets with the highest TREAT‐AD risk scores (i.e., top‐ranked targets). Our analyses consider which targets are common to both lists, as well as the biological functions and target development levels (TDLs) of the targets in each list. We conclude by considering the functional consequences of perturbing targets from each list in disease‐relevant cellular models. We find evidence of a limited overlap between the targets of agents under clinical investigation and those with strong evidence of disease risk association from large‐scale, unbiased assessments of patient data. Part of this difference is simply timing, as campaigns to engage targets that are currently under clinical investigation were started many years ago. Yet even accounting for this lag, there are many risk‐implicated targets for which experimental resources and functional characterization remain limited. This highlights opportunities for concerted efforts to mitigate these challenges and enable therapeutic development around such targets. The Emory‐Sage‐Structural Genomics Consortium (SGC)‐Jackson Laboratory (JAX) TREAT‐AD center is working to generate shared resources and functional data to support therapeutic development around targets prioritized, in part, by these metrics of risk.

## 2 METHODS

### 2.1 Clinical trial target lists

We first identified targets of agents in clinical trials for AD. We considered two sources of information for AD clinical trial data. Over the past decade, Cummings et al. have provided an annual assessment of agents in the clinical trial pipeline for AD,^2^, ^5^, ^6^, ^7^, ^8^, ^9^, ^10^, ^11^, ^12^, ^13^ hereafter called the CADRO pipeline set, and altogether identified 587 distinct trials (i.e., National Clinical Trial [NCT] numbers). As an additional list of AD clinical trials, we also obtained the list of “known drugs” from the Open Targets Application Programming Interface (API).^14^ Open Targets identifies drugs for which there is either a clinical precedent for investigation or approved for use in AD with a mechanism of action curated from the literature. The data obtained from the Open Targets API (accessed October 1, 2025) consists of information pertaining to 503 distinct trials. Overall, there are 159 NCT numbers that are common to both lists (27% to 32% of each list) (Figure S1A). Overall, the trials from the CADRO pipeline papers are more recent, while the trials captured from the Open Targets API are older (Figure S1B). The limited overlap and the disparate date ranges support considering both sources.

We queried the clinicaltrials.gov API for up‐to‐date trial information for all identified NCT numbers. For each study, we captured the trial phase, overall trial status, relevant start and completion dates, and, importantly, the agent under investigation for each trial. Among the accumulated trial information, there were trials of 692 unique agent names for agents in the drug or biological categories. Many of these consist of redundancies (e.g., Donepezil hydrochloride, Donepezil HCL, donepezil HCL). We harmonized agent names by removing common salts (e.g., hydrochloride, sulfate, sodium), removing formulation terms (e.g., tablet, capsule, transdermal), and removing unit or dosage information (e.g., mg, mcg, iu). We also tried to normalize drug names, where possible, using RxNorm.^15^ This left us with 309 unique harmonized drug agent names, 80 of which are common to both sources (Figure S1C).

Molecular targets are known for many of the agents under investigation. To identify these targets, we used the harmonized agent name and queried the PubChem^16^ to retrieve the International Chemical Identifier (InChIKey). We then queried the ChEMBL database^17^ for each unambiguous InChlKey or, for cases where InChlKey was not identified, the harmonized agent name to retrieve the agent mechanism of action, including, where known, the target. We mapped the ChEMBL target identifier to HUGO Gene Nomenclature Committee (HGNC) symbols for integration with downstream processes. In total, we were able to positively identify 271 molecular targets of agents that have been in clinical trials for AD from these two sources (Figure S1D). Agents with no identified molecular targets include cellular and tissue therapies (e.g., Amniotic and Umbilical Cord Tissue, Plasma Exchange, Recombinant Human Serum Albumin), vaccines (e.g., Bacillus Calmette‐Guérin vaccine, Tdap vaccine), natural supplements (e.g., 2‐HOBA, astragalus, curcumin, elderberry juice, Wujia Yizhi), or other experimental interventions (e.g., Fecal Microbiota Transplant, probiotic blend capsule).

### 2.2 Drug‐level Gene Ontology term overrepresentation tests

We tested whether Gene Ontology (GO) Biological Process (BP) terms were targeted by more drugs than expected by chance, while controlling for polypharmacology. GO BP tables mapping GO terms to HGNC gene symbols were generated using the AnnotationDbi version 1.68.0^18^ and org.Hs.eg.db version 3.20.0^19^ R packages. The universe comprises all symbols present in the GO BP mapping. We collapsed multiple drug hits to a single indicator so a drug counted at most once per term, even if it hit multiple targets. This prevented inflation from drugs that hit many subunits within a larger complex (e.g., metformin, a drug used in the treatment of type 2 diabetes, which is thought to target complex I of the mitochondrial electron transport chain) (Figure S2). For each GO term t, the observed statistic is the number of distinct drugs with ≥1 hit in t:

$$O_{t}=\#\left\{drugs\;with\;at\;least\;one\;target\;in\;t\right\}$$

We calculated the observed odds, with Haldane–Anscombe correction (ε=0.5) preventing infinite odds ratios and division‐by‐zero errors when counts were zero, where N is the number of drugs in the set:

$${\mathrm{odds}}_{t}^{\mathrm{obs}}=\frac{O_{t}+0.5}{N-O_{t}+0.5}$$

We generated an empirical null by randomly permuting target labels across all drug–target edges without replacement, repeating B=4000 times. These preserved (i) each drug's number of targets (drug degree) and (ii) each target's overall popularity (target degree), while destroying specific drug→term structures. For each permutation b, we recomputed Ot(b) and computed the permuted odds:

$${\mathrm{odds}}_{t}^{\left(b\right)}=\frac{O_{t}^{\left(b\right)}+0.5}{N-O_{t}^{\left(b\right)}+0.5}$$

We computed a permutation‐relative odds ratio (OR) by taking the median across permutations of:

$${\mathrm{OR}}_{t}^{\mathrm{rel},(b)}=\frac{{\mathrm{odds}}_{t}^{\mathrm{obs}}}{{\mathrm{odds}}_{t}^{\left(b\right)}}$$

One‐sided enrichment *p* values (with +1 correction) were calculated:

$$p_{t}=\frac{1+\sum_{b=1}^{B}1\left\{O_{t}^{\left(b\right)}\geq O_{t}\right\}}{B+1}$$

We controlled false discovery rate (FDR) across terms using Benjamini–Hochberg.^20^

### 2.3 Risk‐implicated targets

We previously described our methodology for systematic assessment of the AD risk of each molecular target across the genome.^4^ Briefly, this involves integrating risk derived from gene association studies (e.g., GWAS, expression quantitative trait loci, and protein quantitative trait loci) with evidence of differential expression of transcripts and proteins based on assessments of *post mortem* brain tissue from the AMP AD program.^21^ AD risk scores were downloaded from the Synapse platform (syn25741025). In total, AD risk association was scored for 24,786 unique genes across the genome. For the basis of comparison we used the highest‐scoring subset of 248 scored targets, to obtain a target set whose size was similar to that of the clinical trial target list, which represents the top 1% of all scored genes. For GO term overrepresentation analysis, we used the enricher function from the clusterProfiler R package version 4.14.6^22^, ^23^ using the same GO BP background as used for the drug term enrichment analyses.

### 2.4 Other metrics

To characterize the druggability of targets on each list, the Pharos TDLs^24^ for each target were downloaded using the provided API.

We also used the list of all targets nominated by the AMP AD program as candidates for new AD treatment or prevention. These targets were nominated based on computational analyses of high‐dimensional data on genomic, proteomic, or metabolomic differences observed in AD from human samples. The current list of nominations, including the data and rationale supporting their consideration, is available on Agora (https://agora.adknowledgeportal.org/genes/nominated‐targets).[Fig alz71142-fig-0001]

We also worked to characterize the functional annotations of each target using GO term annotations grouped into AD‐relevant biological domains.^4^ These biological domain (biodomain) annotations were downloaded from the Synapse platform (syn25428992). Of course, because of the complexity and interrelatedness of many physiological processes and pathways, each gene may be annotated to GO terms resident in multiple biological domains. To assign the top biological domain for each gene, we used the maximum of the sum of three quantities for each term to which the gene is annotated: (1) the fraction of all terms from each domain to which the gene is annotated, (2) the inverse rank of the number of genes annotated to each term, and (3) the rank of the node degree for a graph of terms to which the gene is annotated, where the edges are defined by the number of shared genes between two terms. Significantly enriched terms (FDR ≤ 0.05) from GO term overrepresentation analyses (both drug‐level and gene‐level) were mapped to AD biological domains using the GO term accession.

Finally, to assess gene expression differences following perturbation experiments, we referred to the CRISPRbrain.org resource.^25^ We used the provided API to download the results of gene expression screens for targets of interest in CRISPRa, CRISPRi screens in induced pluripotent stem cell (iPSC)‐derived neurons, astrocytes, and microglia. We correlated log fold change values following target perturbation with the transcriptomic meta‐analysis log fold change values computed as part of the TREAT‐AD risk scoring pipeline.^4^

## 3 RESULTS

### 3.1 Limited overlap between targets in clinical trials and targets with top AD target scores

**FIGURE 1 alz71142-fig-0001:**
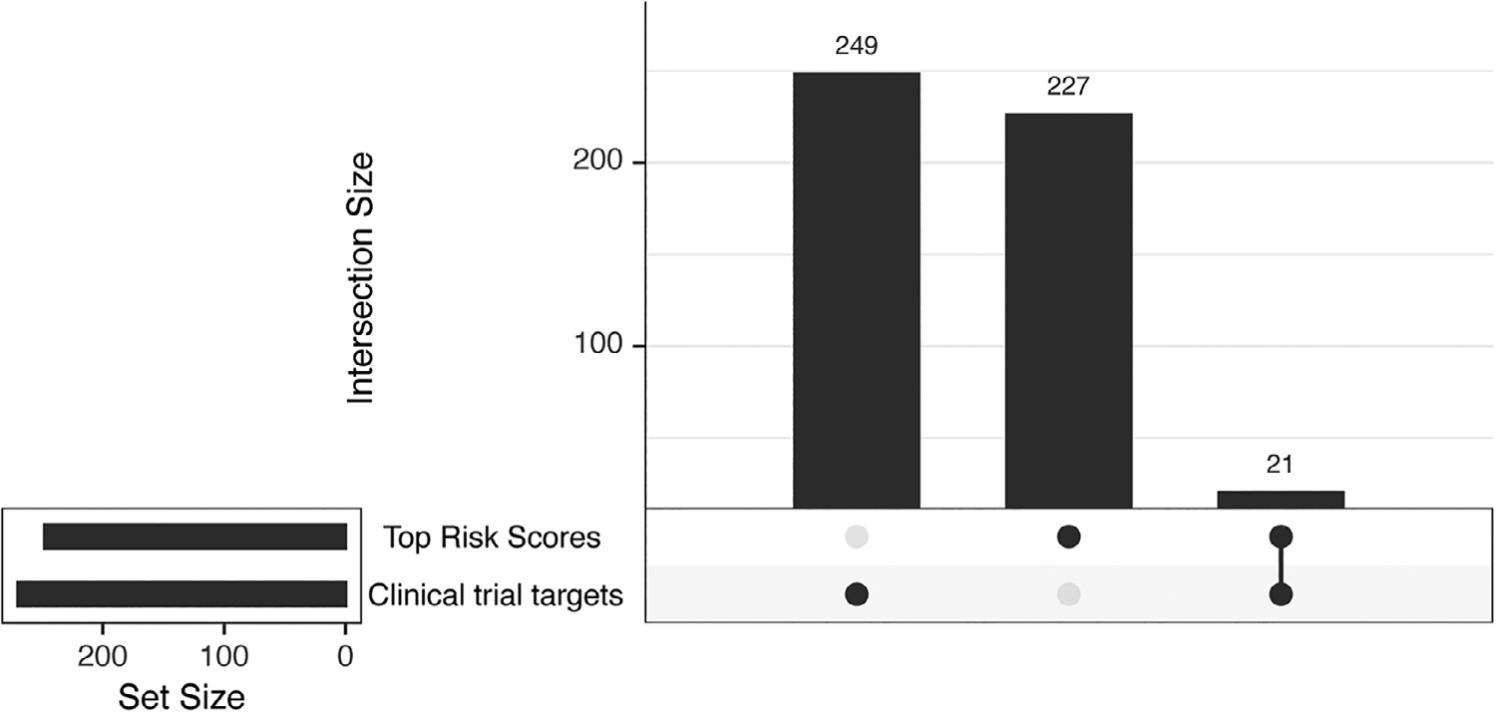
UpSet plot showing the overlap between the list of identified targets of agents in clinical trials for AD and the targets with the top 1% of TREAT‐AD target risk scores.

Of the 271 targets of agents that have been in clinical trials, 21 (7.7%) are among the top 1% of TREAT‐AD Target Risk Scores (TRSs) (Figure 1). This is a significant enrichment (Fisher's exact test *p* = 3.45 × 10^−11^; OR 7.13), which indicates that clinical trial agents are more likely than expected by chance to target genes with strong risk association.

The top‐scoring targets that are currently under clinical investigation are shown in Table 1. Of these targets, two are targeted by more than five distinct agents across trials, including amyloid precursor protein (APP) (36 agents; e.g., aducanumab, donanemab, lecanemab) and microtubule‐associated protein tau (MAPT) (17 agents; e.g., tilavonemab, zagotenemab, semorinemab). The others are targeted by one to four distinct agents across trials with insulin receptor (INSR, four agents; insulin variants), NMDA receptor subunit 2A (GRIN2A, four agents; memantine, neramexane, and dextromethorphan), succinic semialdehyde dehydrogenase involved in GABA metabolism (ALDH5A1, three agents; e.g., valproate, including both valproic acid and divalproex, representing different formulations of the same active pharmacological entity), and the GABA receptor alpha2 subunit (GABRA2, three agents; lorazepam, allopregnanolone, and ac‐3933) having the largest number of distinct agents. Eight of these overlapping targets are part of the mitochondrial electron transport chain (i.e., NDUFS2, NDUFA10, NDUFA9, NDUFS4, NDUFA6, NDUFAF1, NDUFA11, and NDUFS1) and are targets of the same interventional agent: metformin. Notably, metformin has been tested in only two clinical trials, a Phase 2 trial to investigate the effects of metformin on fluid biomarkers (NCT01965756^26^) and an ongoing Phase 2/3 trial expected to be completed in 2026 (NCT04098666). In particular, the number of trials underscores the breadth of coverage for targets directly implicated by the core pathologies of AD (i.e., APP and MAPT) or approved drugs (e.g., memantine for GRIN2A and GRIN2B), while other top‐scoring targets are only targeted by one agent or only tested in one trial. Additionally, 10 of these overlapping targets have been nominated by AMP AD investigators for additional therapeutic development, while the others have not received nominations, despite high risk scores. This is likely due to both the breadth of coverage for some targets (e.g., APP and MAPT), diminishing the need for further development, and the intractability or poorly understood biology of other targets, discussed further in what follows. When we compared the CADRO classification of these overlapping targets with the top TREAT‐AD biological domains for each target, there was good agreement across all targets. This reinforces the utility of the TREAT‐AD biodomains as a useful and largely automated target categorization system, as was the intent.^4^

**TABLE 1:** Table showing the 21 clinical trial targets that are also among the top 1% of TREAT‐AD target risk scores. The columns show the number of unique trials and the number of interventional agents trialed for each target, whether the target has been nominated by AMP AD investigators, as well as the CADRO classification and top AD biodomain.

Gene symbol	No. trials	Trial agents	No. agents	AMP AD nominated	TRS rank (percentile)	CADRO	Top biodomain
APP	80	Solanezumab, aducanumab, lecanemab, donanemab, gantenerumab, and others	36	No	2 (100%)	Anti‐amyloid	APP metabolism
APOE	2	lx1001	1	Yes	3 (100%)	ApoE, lipids, and lipoprotein receptors	Lipid metabolism
NDUFS2	2	Metformin	1	Yes	12 (100%)	Metabolism and bioenergetics	Mitochondrial metabolism
NDUFA10	2		1	Yes	25 (100%)	Metabolism and bioenergetics	Mitochondrial metabolism
NDUFA9	2		1	Yes	71 (100%)	Metabolism and bioenergetics	Mitochondrial metabolism
NDUFS4	2		1	No	81 (100%)	Metabolism and bioenergetics	Mitochondrial metabolism
NDUFA6	2		1	No	128 (99%)	Metabolism and bioenergetics	Mitochondrial metabolism
NDUFAF1	2		1	No	137 (99%)	Metabolism and bioenergetics	Mitochondrial metabolism
NDUFA11	2		1	No	151 (99%)	Metabolism and bioenergetics	Mitochondrial metabolism
NDUFS1	2		1	Yes	182 (99%)	Metabolism and bioenergetics	Mitochondrial metabolism
ALDH5A1	3	Valproate, divalproex, valproic acid	3	No	18 (100%)		Synapse
GRIN2B	15	Memantine, neramexane, ckd‐355a d797/memantine	3	Yes	23 (100%)	Neurotransmitter receptors	Synapse
GRIN2A	17	Memantine, neramexane, ckd‐355a d797/memantine, axs‐05 dextromethorphan‐bupropion	4	Yes	70 (100%)	Neurotransmitter receptors	Synapse
MAPT	19	Tilavonemab, zagotenemab, semorinemab, bepranemab, ly3372689, e2814, …	17	No	30 (100%)	Anti‐tau	Tau homeostasis
TREM2	2	al002	1	Yes	37 (100%)	Inflammation	Immune response
GABBR1	1	sgs742	1	No	93 (100%)		Synapse
GABRA2	5	Allopregnanolone, ac‐3933, lorazepam	3	Yes	187 (99%)	Neurogenesis	Synapse
ABCA1	2	Probucol, cs6253	2	No	188 (99%)	APOE, lipids and lipoprotein receptors	Lipid metabolism
INSR	6	Insulin glulisine, insulin aspart, insulin, insulin + empagliflozin	4	Yes	214 (99%)	Metabolism and bioenergetics	APP metabolism
RAC1	1	50561	1	No	224 (99%)	Synaptic Plasticity/neuroprotection	Mitochondrial metabolism
SEMA4D	1	Pepinemab	1	No	239 (99%)	Inflammation	Immune response

Abbreviations: AMP AD, Accelerating Medicines Partnership Program for Alzheimer's Disease; apoE, apolipoprotein E; APP, amyloid precursor protein; CADRO, Common Alzheimer's Disease Research Ontology; TRS, Target Risk Score.

### 3.2 Contrasting biological functions and target development maturity across AD target lists

**FIGURE 2:**
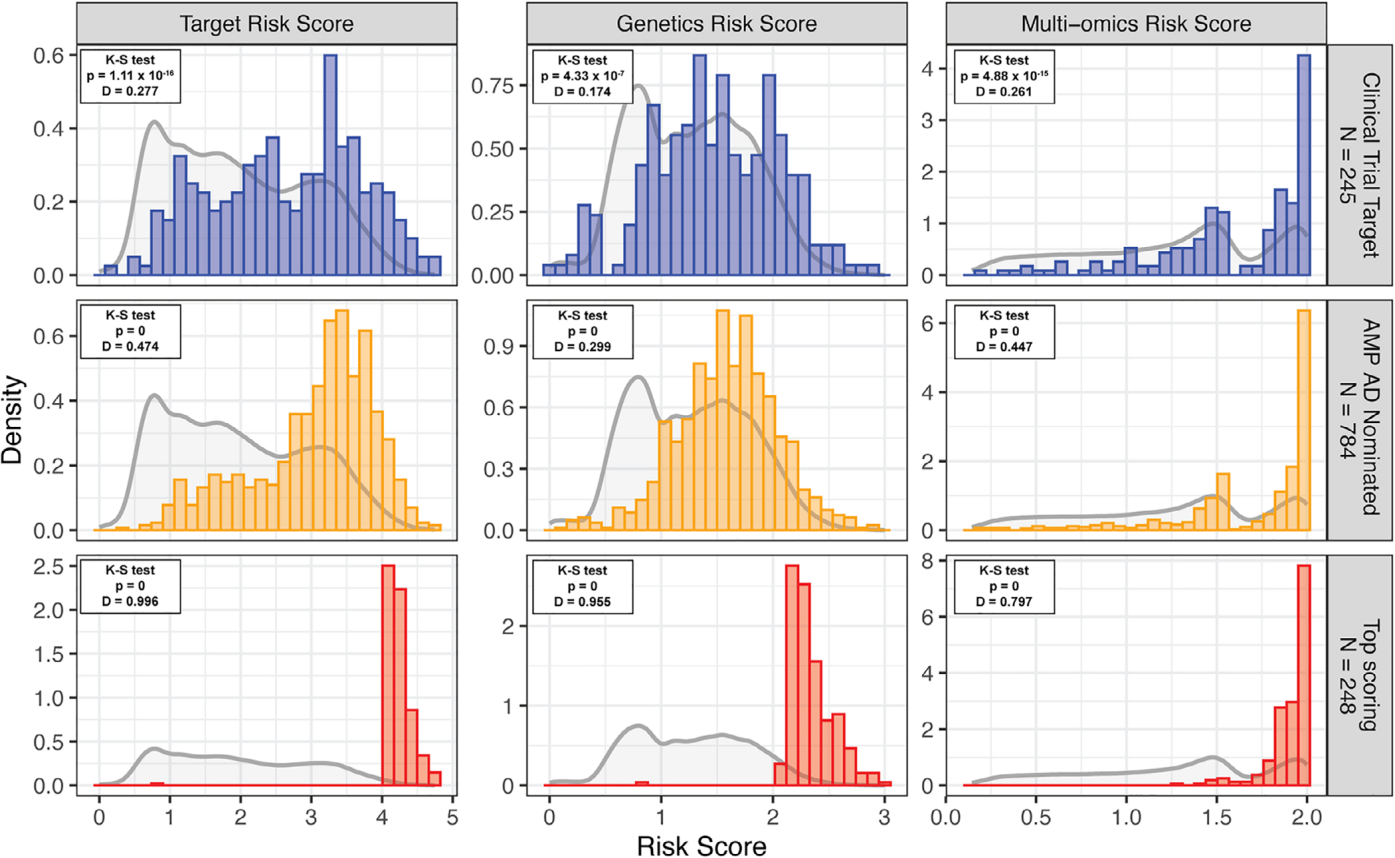
TREAT‐AD Target Risk Scores and component (i.e., Genetics and Multi‐Omics) scores for targets in different classes. For each, the background distribution of all scored genes is shown as a grey line, and the histogram shows the distribution of targets in that category. The Kolmogorov‐Smirnov (K‐S) test statistics are shown for each distribution versus the background distribution.

To more comprehensively contrast the targets within each list, we started by comparing the TREAT‐AD TRS distributions for each target list (Figure 2). Given that the top‐scoring set is drawn from the top 1%, the distribution of the scores for these targets is heavily skewed to the upper end of the distribution (K‐S test *p* = 0 for all risk scores). Likewise, the risk scores for the set of AMP AD‐nominated targets are also significantly skewed to the upper end of the distribution (K‐S test *p* = 0 for all risk scores), reflecting that the risk scores are derived from AMP AD gene and protein expression data. Comparing the distribution of AD risk scores for clinical trial targets to the distribution of all other genes, we note that the risk scores are significantly higher for the trial targets. The TRS distribution for trial targets is higher than for non‐targeted proteins (K‐S test *p* = 1.11 × 10^−16^, *D* = 0.28), as are the distributions for the genetic risk score (K‐S test *p* = 4.33 × 10^−7^, D = 0.17) and multi‐omic risk score (K‐S test *p* = 4.88 × 10^−15^, *D* = 0.26). Of note, for both the clinical trial target and AMP AD‐nominated target distributions are more skewed for the multi‐omics risk score (*D* = 0.261 and 0.447, respectively) than the genetics risk score (*D* = 0.174 and 0.299, respectively), while the top‐scoring targets have a relatively higher skew for the genetics risk score (*D* = 0.955) than for the multi‐omics risk score (*D* = 0.797). This is consistent with the multi‐omics risk scores drawing heavily from the AMP AD datasets, but it is important given the observed impact of targets with genetic support being over twice as successful in clinical trials.^27^ Overall, these comparisons indicate that targets selected for clinical development are not arbitrary; they carry a significantly higher burden of risk than the average gene, providing a crucial baseline for further comparison.

**FIGURE 3:**
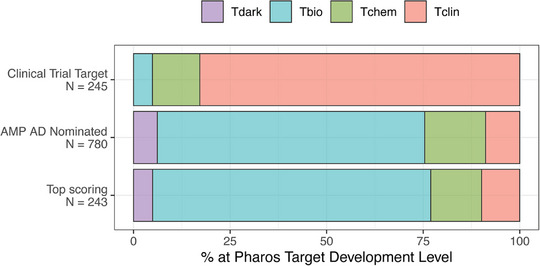
Proportions of each target set in each Pharos target development level (TDL). The levels are defined where: Tclin targets have an approved drug, Tchem targets have known small molecule binders, Tbio targets do not have any small molecules but where functions have been characterized, and Tdark targets are those about which virtually nothing is known.

There is also a sharp contrast between the lists when considering the developmental maturity of the targets in each list (Figure 3). For this comparison, we utilized the Pharos TDLs to characterize the amount of data available about each target, which could be used to inform therapeutic development. There are four TDLs: Tclin, Tchem, Tbio, and Tdark. Targets that are classified as Tclin have at least one approved drug, whereas Tchem has at least one ChEMBL compound with an activity less than 30 nM. Tbio targets do not have any known drugs or small‐molecule activities, and Tdark targets are those about which virtually nothing is known. The clinical trial targets have proportionally higher representation among Tclin proteins (83%) than the AMP AD‐nominated (8.8%) or top‐scoring targets (9.9%). Given that a target must have a therapeutic agent to enter a trial, this skew toward Tclin is expected; however, it quantifies a skew toward therapeutic development around well‐characterized biology rather than less explored mechanisms. Conversely, the top‐scoring (72%) and AMP AD‐nominated (69%) targets are proportionally more represented among the Tbio classification, with no small molecules yet available, whereas the vast majority of clinical trial targets are already at mature stages of drug development (only 4.9% without any known drugs). Furthermore, there are 12 targets among the top‐scoring set that are classified as dark targets with little or no understanding of their biology (Tdark, 4.9%) and a similar proportion among AMP AD‐nominated targets (6.2%), while, as expected, there are no dark targets among the clinical trial set. The proportion of targets in Tchem with high‐affinity reagents is equivalent between the sets (12% for trial targets, 16% for AMP AD‐nominated targets, and 13% for top‐scoring targets). Overall, this demonstrates a “streetlight effect” in AD therapeutic development – targets under clinical investigation for AD represent well‐known and developed targets, while the top risk‐implicated targets are proportionally more likely to fall into the “dark” category, reflecting limited biological characterization.

**FIGURE 4:**
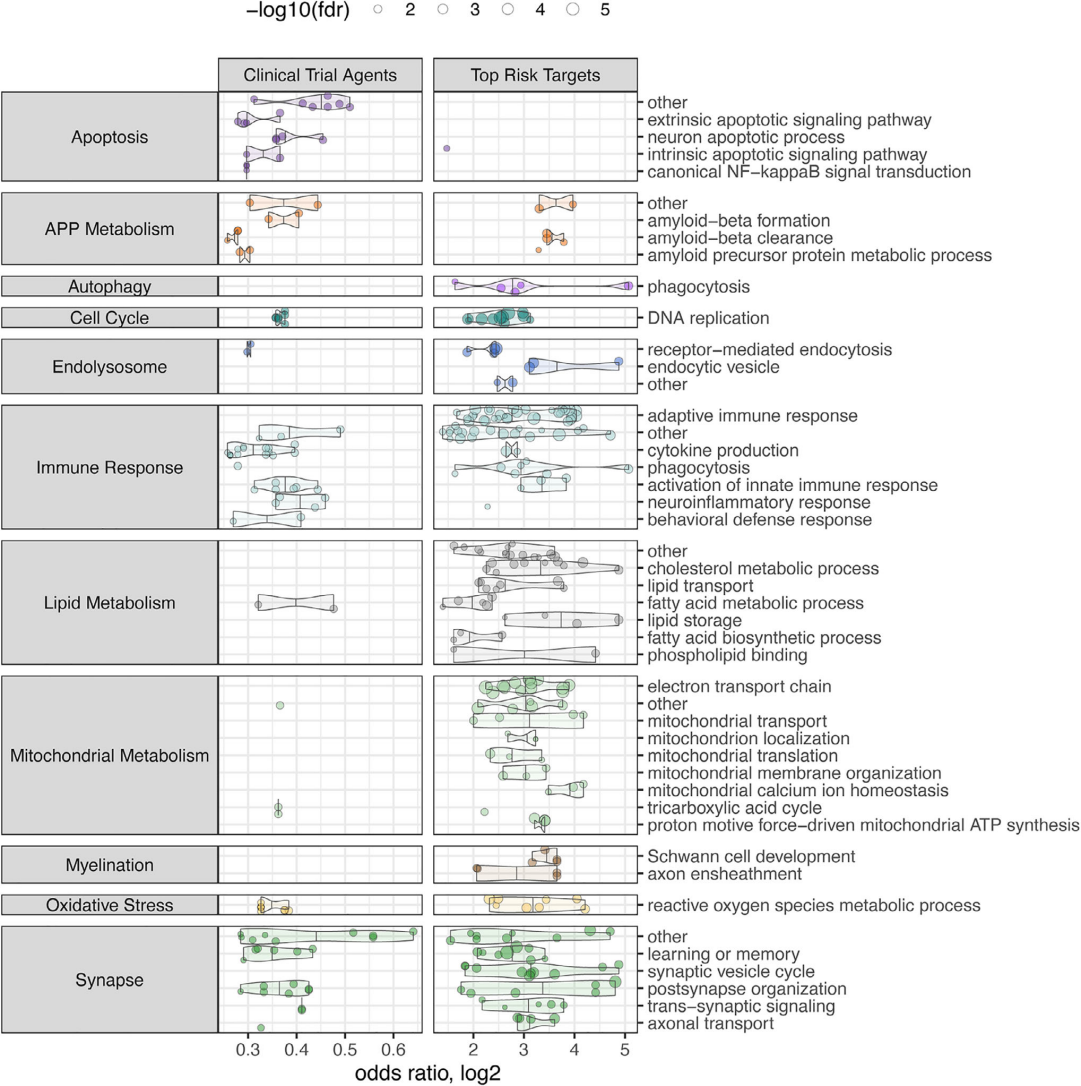
Overrepresented AD subdomain terms for clinical trial agents and targets with top risk scores. For each target set, the plot shows significantly enriched GO terms (i.e., FDR ≤ 0.05) from each AD subdomain (*y*‐axis) within a parent domain (left facet). The size of each point corresponds to the FDR significance (−log_10_) of the enrichment, and the position on the *x*‐axis corresponds to the log_2_ odds ratio of the observed enrichment.

Finally, we characterized the functional annotations of each target list using GO term overrepresentation tests and then mapped significantly enriched terms onto the AD biodomains. We noted that many of the agents in clinical trials had more than two identified molecular targets, including nine agents with at least 10 distinct molecular targets identified, with metformin having 54 identified molecular targets (Figure S2), predominantly protein subunits of complex I from the mitochondrial electron transport chain. To mitigate the effects of this polypharmacology from biasing the functional enrichments, we used the bipartite graph of clinical trial agents and identified targets to map agents to biological process terms from the GO and used these mappings to test for overrepresentation among GO terms. We mapped significantly enriched (FDR ≤ 0.05) GO terms onto the TREAT‐AD biological domains (biodomain) (Figure 4). The clinical trial agents are more overrepresented among terms from the Apoptosis biodomain than the targets with the top risk scores. Conversely, the top‐scoring targets are more overrepresented among GO terms within the Mitochondrial Metabolism, Myelination, Lipid Metabolism, and Autophagy biodomains. Even for biodomains where terms are enriched for both sets (e.g., APP metabolism, synapse, immune response, and endolysosome), when we examined the subdomain classifications of overrepresented terms, we noted some key differences (Cary et al., current issue). APP metabolism has been the primary focus of AD drug development, and while there are enrichments for both sets of terms from the “amyloid beta clearance” subdomain, the clinical trial agents are more enriched among the “amyloid beta formation” and “amyloid precursor protein metabolic process” subdomains. Though there are similar enrichments for both lists within the Synapse domain (e.g., for terms from “learning and memory” and “postsynapse organization”), terms from the “synaptic vesicle cycle” are uniquely overrepresented among the top‐scoring targets. Likewise, for the Endolysosome domain, both sets are enriched for terms from the “receptor‐mediated endocytosis” subdomain, while enrichments from the “endocytic vesicle” subdomain are unique to the top‐scoring set. Finally, for the Immune Response biodomain, there are common enrichments for terms from the “cytokine production” and “activation of innate immune response” subdomains, while the enrichments for terms in the “neuroinflammatory response” and “behavioral defense response” subdomains are unique to the clinical trial agents and “adaptive immune response” and “phagocytosis” subdomains are unique to the top scoring targets. Overall, the agents in clinical trials for AD emphasize apoptotic and neuroinflammatory processes, while the risk‐implicated targets highlight mitochondrial metabolism, lipid and myelin biology, and autophagy. This underscores a strategic divergence and suggests that incorporating additional disease‐associated biology into therapeutic portfolios may represent an opportunity to improve translational success.

### 3.3 Assessing transcriptomic reversal potential for AD targets

**FIGURE 5:**
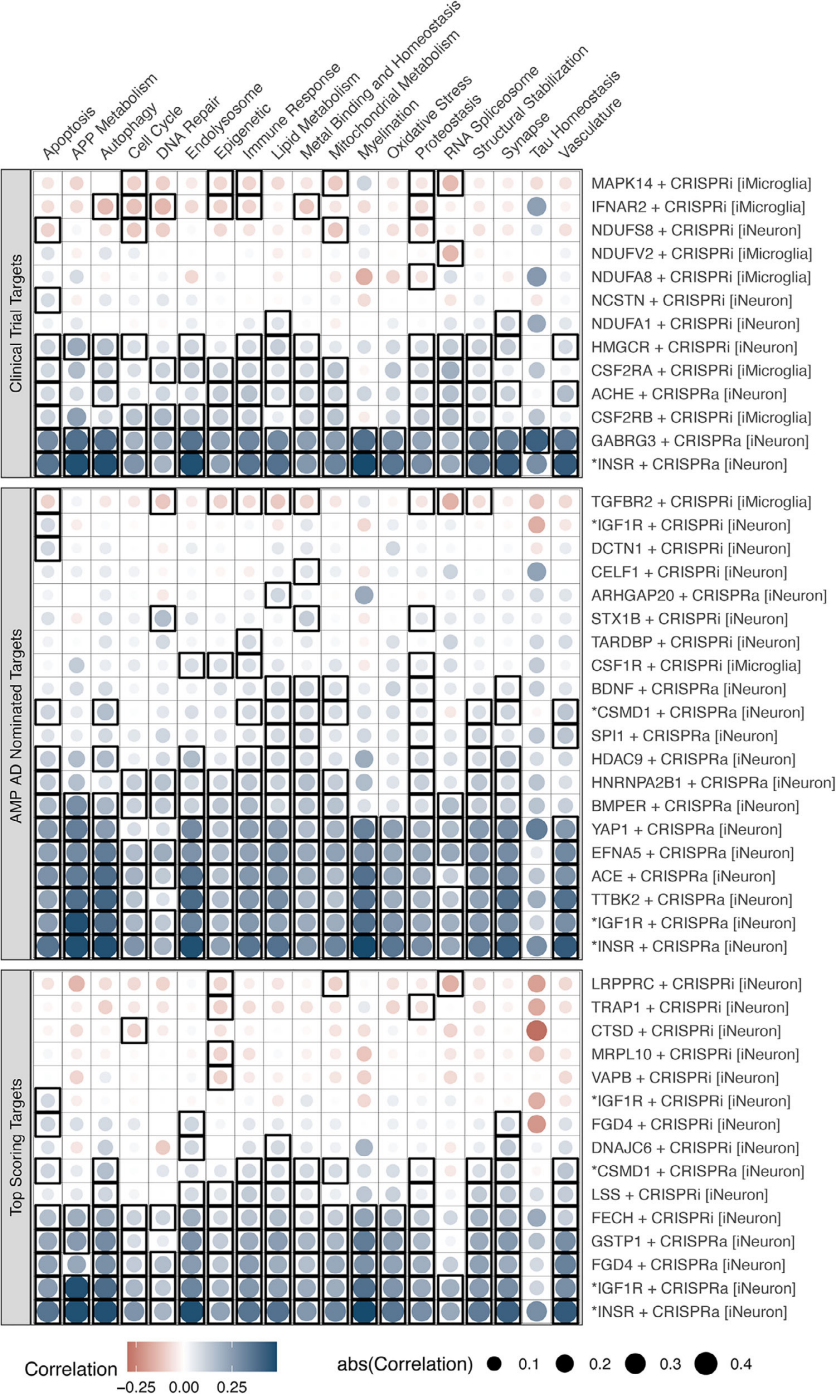
Transcriptome correlations between CRISPR perturbed iPSC cells and meta‐analysis results of AMP AD datasets. For each target perturbation (*y*‐axis), the results of correlations for genes within each AD biodomain (*x*‐axis) are shown. The fill color of each point represents the Pearson correlation coefficient, while the size of each point shows the magnitude of the correlation. Correlations that were significant after adjusting for multiple hypothesis testing are outlined with a black square.

Optimal therapeutic targets will have the capacity to counteract disease‐associated processes when perturbed. To assess the functional consequences of perturbing targets from each set, we utilized data from high‐throughput CRISPR screens performed in iPSC‐derived cell lines from the CRISPRbrain resource (crisprbrain.org^25^). These data characterize the transcriptomic response to increasing expression (CRISPRa) or decreasing expression (CRISPRi) of a given target gene in human iPSC‐derived brain‐relevant cell types (i.e., neurons and microglia). So far, CRISPRbrain contains RNA‐seq data following genetic perturbation of 35 of the identified AMP AD‐nominated targets, 25 of the top‐scoring targets, and 22 of the clinical trial targets. This gives us an opportunity to consider the extent to which perturbation of targets from these different sets significantly alters the transcriptome relative to AD‐relevant changes. We correlated gene expression changes following CRISPR perturbations with the results of our transcriptomic meta‐analysis of AD.^4^ These analyses, stratified by AD biodomain, highlight which perturbations induce cell expression profiles toward (i.e., positive correlation) or away from (i.e., negative correlation) the transcriptomic signatures present in *post mortem* AD brain data from AMP AD (Figure 5). We emphasize that genetic perturbation via CRISPRa or CRISPRi does not model pharmacological activation or inhibition but rather provides a controlled means of assessing the transcriptomic consequences of increasing or decreasing gene expression.

As an internal sanity check for this approach, we first assessed perturbation of acetylcholinesterase (ACHE), given that this is a well‐established target for several US Food and Drug Administration (FDA)‐approved drugs for treating AD. Increasing the expression of ACHE with CRISPRa in iNeurons produced a modest but consistent positive correlation with disease signatures in 12 of 19 biodomains. Given that ACHE inhibition results in the desired therapeutic effects, the observation that activation of ACHE produces a positive correlation to AD brain expression profiles implies that inhibition would be predicted to have the opposite, disease‐reversing effect. Supporting this inference, we note that while activation of IGF1R in neurons resulted in positive correlation with AD transcriptome signatures across 18 biodomains, inhibition of the same target only has a significant positive correlation for one biodomain, and several other biodomain correlations are negative, though not significant. Considering the results of other targets from clinical trials, we note that activation of the insulin receptor (INSR) in iNeurons resulted in a significant and strong positive correlation across 18 of 19 domains, which contradicts the therapeutic hypothesis around this target that boosting insulin signaling would be broadly beneficial in AD. The therapeutic effects of agonizing INSR signaling may be more nuanced in vivo, and simply increasing the expression level of the receptor may lead to negative feedback regulation through insulin receptor substrate protein phosphorylation and receptor endocytosis.^28^ These observations should not be interpreted as predictive of clinical efficacy, as receptor signaling dynamics, dosage, and tissue‐level feedback mechanisms in vivo are not captured by this perturbation paradigm. Five of the clinical trial targets analyzed in the CRISPRbrain data (MAPK14, IFNAR2, NDUFS8, NDUFV2, and NDUFA8) resulted in significant anti‐correlation with disease signatures across at least one biodomain. Of the AMP AD‐nominated targets, only TGFBR2 inhibition in microglia resulted in anti‐correlation with AD transcriptomes. Five of the perturbations of risk‐implicated targets (LRPPRC, TRAP1, CTSD, MRPL10, and VAPB) resulted in negative correlation with AD expression profiles. Hence, this represents a useful early‐stage approach for screening candidate targets for engagement and effectiveness during preclinical assessment and reflects our approach to validating nominated targets within the TREAT‐AD consortium.

## 4 DISCUSSION

Therapeutic development for AD remains concentrated under the streetlight within a relatively small set of well‐characterized targets, largely focused on amyloid, tau, and a limited number of additional biological pathways. Recent systems‐level evidence from large‐scale human genetic and multi‐omics studies has highlighted additional disease‐associated biology beyond these well‐established areas. Many of these implicated targets remain challenging to pursue, given limited biological characterization and a scarcity of experimental reagents to support therapeutic development. In this context, the role of the National Institute on Aging (NIA)‐funded TREAT‐AD centers are to support the systematic enablement of such targets by generating shared resources and functional data that can inform future development efforts. These activities emphasize integrative analyses of human cohort data to prioritize targets, open generation of experimental tools, and evaluation of functional consequences in relevant model systems.

Using tools developed by TREAT‐AD, we characterized the AD clinical trial landscape and examined how it relates to biology implicated by human genetic and multi‐omic risk assessments. We observe a limited but non‐random overlap between clinical trial targets and the top 1% of AD risk scores and note that even clinical trial targets outside this top tier exhibit an elevated risk score distribution. As expected, there are marked differences in the target development maturity between targets under clinical investigation and the larger set of targets more recently implicated by systems‐level analyses of disease. Clinical trials are, by necessity, enriched for targets with existing therapeutic agents (i.e., Tclin and Tchem targets), whereas many risk‐implicated targets fall within the Tbio or Tdark development levels, where substantial biological characterization and tool development remain necessary. FDA‐approved drugs account for a substantial number of clinical trials, including agents approved for AD (e.g., memantine) as well as those approved for indications other than AD (e.g., metformin), reflecting a pragmatic focus on well‐established targets. At the same time, the definition of what constitutes an actionable or “druggable” target continues to evolve with the adoption of new molecular and genetic interventions (e.g., anti‐sense oligonucleotides). Beyond differences in development maturity, we also observe functional distinctions between the two target sets. Clinical trial agents are overrepresented for processes from the APP Metabolism, Synapse (reflecting neurotransmitter therapeutics), Immune Response, and Apoptosis biodomains. The prominence of certain mechanisms being tested in clinical trials reflects not only biological relevance but also evolving scientific narratives, prior trial outcomes, reputational dynamics, and business priorities. As a result, entire classes of mechanisms may be emphasized or deprioritized over time, independent of their underlying involvement in disease. In contrast, many top‐risk targets show enrichment for Mitochondrial Metabolism, Lipid Metabolism, Myelination, and Autophagy biodomains, for which there is comparatively little enrichment among trial targets. For example, only two trials target the top‐risk components from complex I of the mitochondrial electron transport chain, both repurposing metformin. These observations highlight areas where disease‐associated biology remains comparatively less explored in the clinical pipeline. Finally, we emphasize that risk association alone is insufficient to prioritize therapeutic targets. Functional screening is essential to identify subsets of targets whose perturbation has the capacity to influence AD‐relevant phenotypes. Some risk‐implicated targets may reflect downstream consequences of pathology or occupy positions within cellular networks that limit their broader functional impact.

The observed differences between risk‐implicated biology and targets undergoing clinical trials are due, in part, to limitations in the availability of experimental resources and data needed to support systematic target evaluation. Resources such as molecular structures, biochemical and cellular assays, validated reagents, chemical matter, and disease‐relevant models are often required for targets to progress toward clinical investigation. The establishment and validation of these resources can be costly and time‐consuming, creating structural incentives to favor developmental paths centered on targets with established biology and existing toolsets. Targets implicated by human risk data but lacking such resources face higher barriers to advancement. Given the biological complexity and heterogeneity of AD and ADRD, addressing these barriers represents an important opportunity to broaden the range of mechanisms that can be rigorously evaluated in therapeutic development.

The approach employed within the Emory‐Sage‐SGC‐JAX TREAT‐AD center is centered around three “tiers” of target development. First, we integrate evidence from human studies to support the nomination of targets for entry into exploratory development efforts. This involves integrating the rich, multimodal evidence from genetics and multi‐omics studies, with AMP AD serving as a cornerstone data resource, to generate target‐level risk metrics. We have also developed the AD biodomains to organize biological processes in an automatable fashion for interpretability and dataset interoperability. The second tier focuses on systematic enablement through target‐specific resource development. This includes building and openly disseminating experimental resources such as validated antibodies, molecular structures, biochemical and cellular assays, and chemical probes or tool biologics. These efforts are intended to reduce technical barriers to studying targets that currently lack sufficient tools, particularly those classified as Tdark or Tbio. Tier 3 of our strategy involves functional interrogation in disease‐relevant model systems. When appropriate enabling resources are available, we examine the effects of target perturbation in human iPSC‐derived neurons, astrocytes, and microglia, as well as in well‐characterized animal models. The emphasis at this stage is on assessing whether and how target engagement influences AD‐relevant phenotypes, including omic‐level responses, and on identifying potential context dependence or off‐target effects. Evidence generated and openly disseminated in this tier can be used to inform subsequent experimental prioritization and translational considerations.

Rather than abandoning well‐lit therapeutic pathways, this framework is intended to broaden consideration of what may constitute an actionable target. Resources developed by the TREAT‐AD program are openly available to support target portfolio design and experimental evaluation. In this way, these efforts may expand exploration of disease‐associated biology that is currently underrepresented in the clinical pipeline (e.g., mitochondrial and lipid metabolism), while also supporting continued investigation of pathways already aligned with risk (e.g., synaptic and immune biology). Over time, increased use of systematic risk metrics could contribute to shifts in the distribution of risk scores represented among targets entering development as risk metrics continue to be enhanced through the inclusion of new datasets. This framework may also be useful for examining biological processes that lie at the intersection of multiple domains, which could represent points of systems‐level interaction or inform combination strategies. Resources generated through this effort could also be used to construct molecular fingerprints – multi‐omic, biodomain‐resolved signatures – that may serve as provisional pharmacodynamic readouts that complement single‐analyte biomarkers, guide biomarker discovery by prioritizing tractable cerebrospinal fluid, plasma, or imaging candidates, and inform aspects of trial design such as patient stratification or dose exploration. Because the biodomains provide an interpretable mapping between mechanisms and phenotypes, they can also aid in assessing model system relevance, evaluating systems‐level impacts on disease‐associated signatures, and identifying potential off‐target impacts. In this way, biodomain‐resolved molecular signatures can provide a structured framework for assessing broader, system‐level consequences of genetic or pharmacologic perturbation, including cascading or unanticipated effects that extend beyond a drug's nominal mechanism of action and may inform interpretation of complex or pleiotropic responses. Collectively, these resources are intended to improve target enablement and support earlier, mechanism‐informed decision‐making during preclinical development, with the goal of identifying targets that demonstrate reproducible, disease‐relevant phenotypic effects. As AMP AD continues to expand data generation across additional cohorts and molecular modalities, the utility of this framework is likely to increase for studies of disease heterogeneity, early intervention, and precision medicine‐oriented approaches.

There are limitations to our assessments here. We aimed to gather all relevant clinical trial data. While OpenTargets is a valuable resource, especially for historical trial information, and the CADRO pipeline papers have done an excellent job cataloging and characterizing recent trials, there may still be gaps in the coverage of trials. We found that accurately mapping targets of clinical trial agents was a non‐trivial task. Agent name harmonization and cross‐database mapping can naturally miss or misalign targets with drugs, which is especially problematic for multicomponent or pleiotropic interventions (e.g., combination therapies or widely repurposed agents such as metformin). Additionally, the timeline of therapeutic development is long, and compounds still in preclinical stages are not considered. There are also limitations to the model systems we queried. While iPSC‐derived neurons and microglia capture cell‐intrinsic programs, there may be non‐cell autonomous functions that would be illuminated through the use of co‐culture systems, *ex vivo* organoid or minibrain approaches, or appropriate animal models.

The TREAT‐AD centers aim to continue evaluating and prioritizing targets for enablement, working in collaboration with the AD research community to consider both internally identified and externally nominated candidates. The centers produce Target Enabling Packages (TEPs), which are openly disseminated and include the experimental tools and resources developed and validated to further the investigation of selected targets. These efforts emphasize targets supported by accumulated evidence from human genetic and molecular studies, including AMP AD and complementary datasets, particularly where limited existing resources have constrained evaluation. By expanding the availability of shared tools and data, this approach seeks to lower barriers to probing disease‐associated biology that has historically received less investigational attention.

## CONFLICT OF INTEREST STATEMENT

G.A.C., J.C.W.: No conflicts of interest. A.I.L. is a paid consultant for EmTheraPro, Cognito Therapeutics, Cognition Therapeutics, and Alamar. G.W.C. is a paid consultant for Astrex Pharmaceuticals. Author disclosures are available in the Supporting Information.

## Supporting information



Supporting Information

Supporting Information
