# Supplementary material for: Beyond the streetlight: a TREAT‐AD perspective on where to find new Alzheimer's targets
Source: Alzheimers Dement. 2026 Feb 13;22(2):e71142. doi: 10.1002/alz.71142 (PMC12902793; doi:10.1002/alz.71142)
Supplement: Supplementary file 1 — Supporting Information [file ALZ-22-e71142-s002.docx]

**Beyond the Streetlight: A TREAT-AD Perspective on Where to Find New Alzheimer's Targets**

—-

**SUPPLEMENTAL FIGURES**

**Supplementary Figure 1**


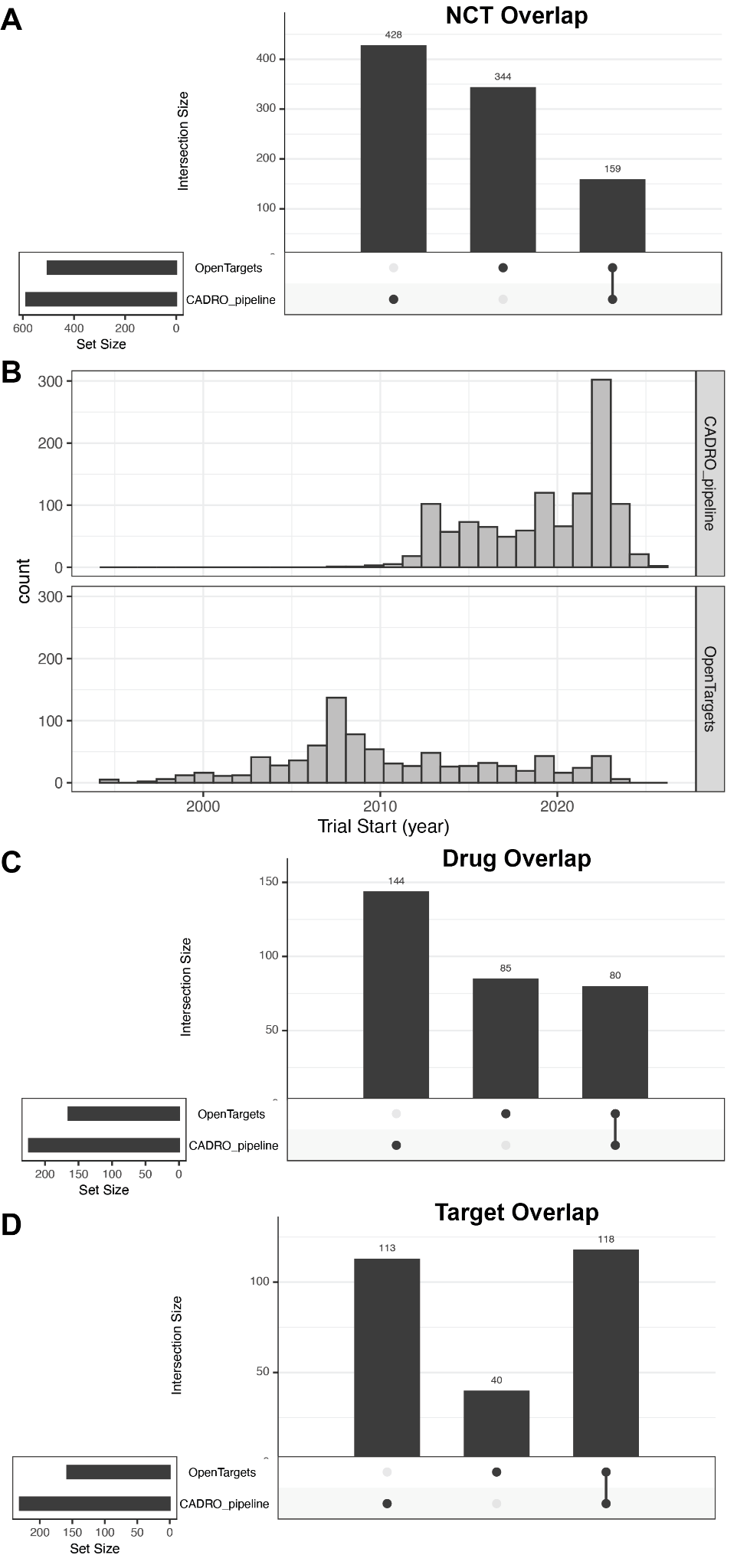


**Supplementary Figure 1.** Comparing CADRO pipeline and OpenTargets sources of AD clinical trial information. (A) UpSet plot showing the overlapping NCT numbers. (B) Trial start year distribution. (C) UpSet plot showing the overlapping drugs or clinical trial agents. (D) UpSet plot showing the overlapping drug targets or proteins identified.

**Supplementary Figure 2**


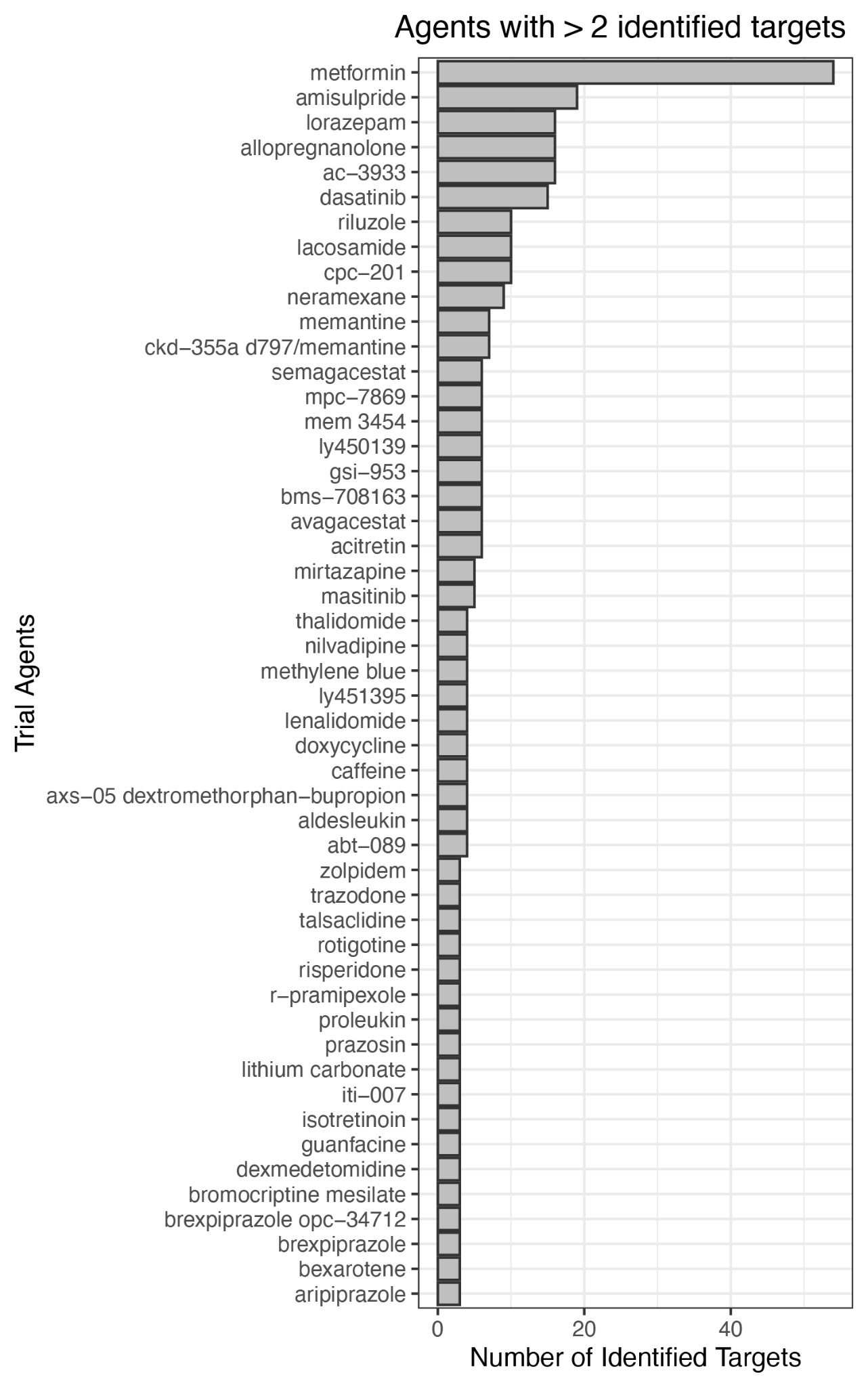


**Supplementary Figure 2.** Characterizing drug polypharmacology. Clinical trial agents (i.e., drugs) with multiple molecular targets identified are shown.
